# Reconstruction of isolated hypoplasia of the patella by a modified Galeazzi procedure: a case report

**DOI:** 10.1186/s13256-023-04122-6

**Published:** 2023-09-09

**Authors:** Mohamed Laroussi Toumia, Ahmed Amine Mohseni, Souha Bennour, Mohamed Nabil Nessib, Rim Boussetta, Sami Bouchoucha

**Affiliations:** Department of Pediatric Orthopaedic Surgery, Children’s Hospital “Bechir HAMZA”, Medical School of Tunis, University Tunis-El Manar, Tunis, Tunisia

**Keywords:** Patella, Hypoplasia, Aplasia, Tendon transfer, Case report

## Abstract

**Background:**

Isolated Patellar Aplasia Hypoplasia is a very rare autosomal dominant disorder. Its treatment depends on the clinical manifestations that can vary widely. The lack of active extension, which can be responsible for frequent falls due to a knee instability, is the most frequent and disabling manifestation. We report an original technique that is a modification of the Galeazzi technique for recurrent dislocation of the patella to gain active extension in case of PTLAH.

**Case report:**

A 7-year-old Caucasian boy with isolated Patellar Aplasia Hypoplasia and an extension lag of the right knee has been treated by a modified Galeazzi technique. The tendons of the semi-tendinous and gracilis muscles have been harvested and their distal insertion was kept intact. Both tendons were fixed over the top of the patella to restore knee active extension. After 6 years of follow up the patient is symptom free with a strong active extension of the operated knee.

**Conclusion:**

Reconstruction of isolated hypoplasia of the patella by a modified Galeazzi procedure is a safe and reliable technique for skeletally immature patients offering satisfying long-term outcomes.

## Introduction

The Patellar Aplasia/hypoplasia (PTLAH) is a symptom present in several syndromes [1] like Nail Patella syndrome where it’s associated with other manifestations such as fingernails dystrophy. Isolated PTLAH is a very rare autosomal dominant disorder with no other manifestation that always affects both knees [2]. Its treatment depends on the clinical manifestations that can vary widely [3]. The lack of active extension which can be responsible for frequent falls due to a knee instability is the most frequent and disabling manifestation.

We report an original technique that is a modification of the Galeazzi technique [4] for recurrent dislocation of the patella to gain active extension in case of PTLAH.

## Case report

A 7-year-old Caucasian boy was referred to our outpatient clinic for frequent falls due to both knees instability that was more pronounced in the right side. The family history revealed the presence of the same symptoms in his father.

On examination, full range of motion of both knees was noted with an extension lag on active motion of 120° for the right side and 40° for the left side.

The patella was not palpated into the patellofemoral groove with a protrusion of the femoral condyles. We could palpate a very small patella on the anterior aspect of the distal third of the thigh well above the patellofemoral groove. No palpable patella at all even on an ectopic position could be identified on the left knee. In both knees the patellar tendon could not be palpated. The patient was not able to hop on either leg. The rest of the clinical examination did not find any other orthopedic anomaly. The fingernails and toenails were normal. The psychomotor development of the child was normal.

**Fig. 1:**
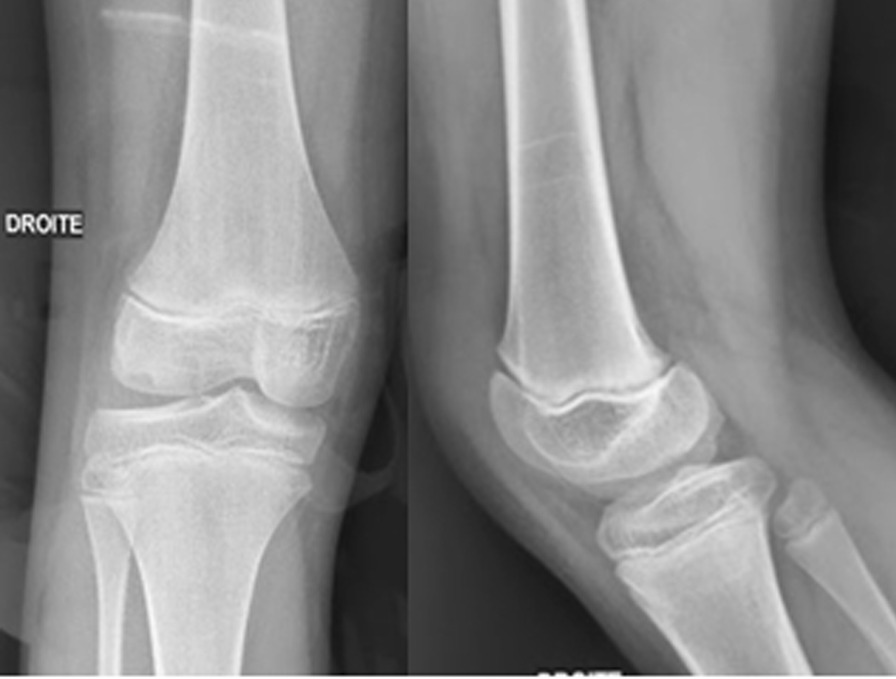
AP and lateral radiographs of the child’s right knee at the last follow up

The patella was not visible on plain AP and lateral radiographs of both knees (Fig. 1).

**Fig. 2:**
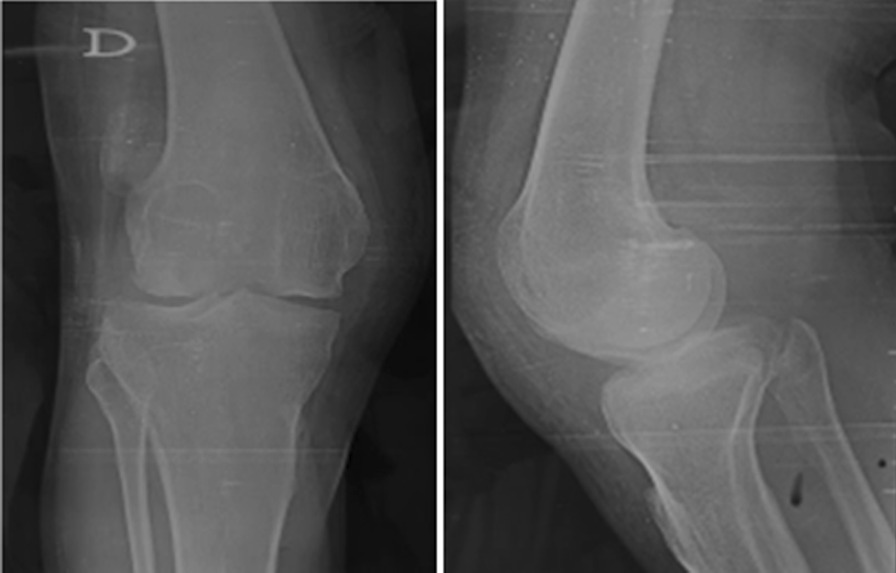
AP and lateral radiographs of the child’s father showing the laterally dislocated hypoplastic patella

The MRI found an ectopic small patella in the right knee but no patella on the left side. The radiographs of the child’s father who was 40 years old showed a bilateral small laterally dislocated patella (Fig. 2).

Regarding the importance of the gait instability, it has been decided to surgically reconstruct the extensor mechanism of the right knee to regain active extension.

### Surgical technique

The surgery was performed with the child in the supine position under a tourniquet hemostasis. With a small oblique incision on the medial aspect of the proximal tibia, we were able to identified the tendons of the semi-tendinous and gracilis muscles. With a tendon stripper we harvested both tendons at the musculotendinous junction and kept their tibial insertion intact.

**Fig. 3:**
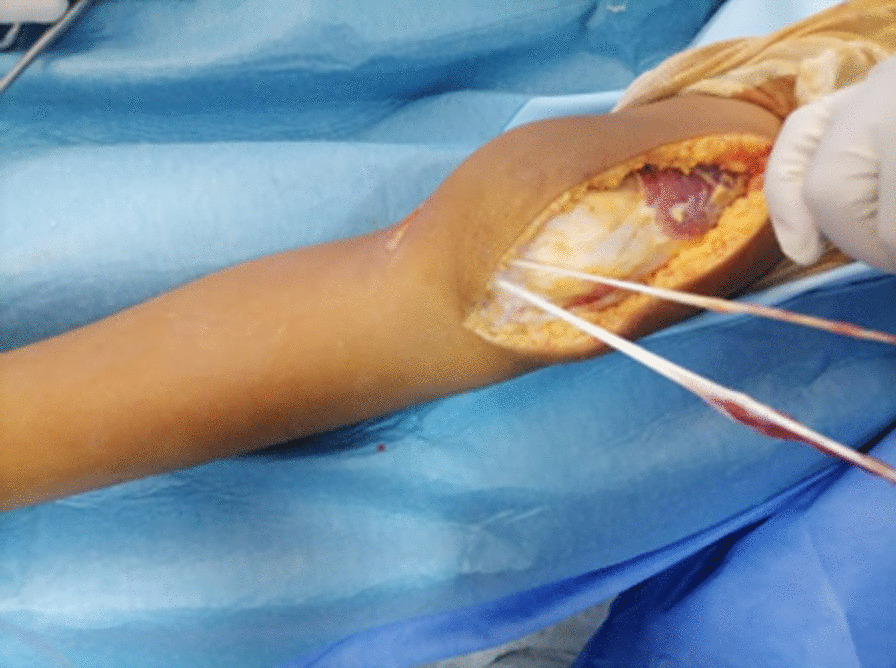
The tendons subcutaneously tunneled and exited through the anterior approach

We then performed an anterior approach of the knee starting from above the small patella in the distal thigh, extending distally to the patellofemoral groove. The patella appeared small and the vestigial patellar tendon consisted of fibrotic bundle. The semi-tendinous and gracilis tendons were subcutaneously tunneled form their tibial insertion (Fig. 3) exited through the anterior approach and sutured together. They were then passed over the top of the hypoplastic patella from the inferomedial to the superolateral corners, folded back over the anterior aspect of the patella and sutured to themselves with tension while the knee was maintained in 30° of flexion.

**Fig. 4:**
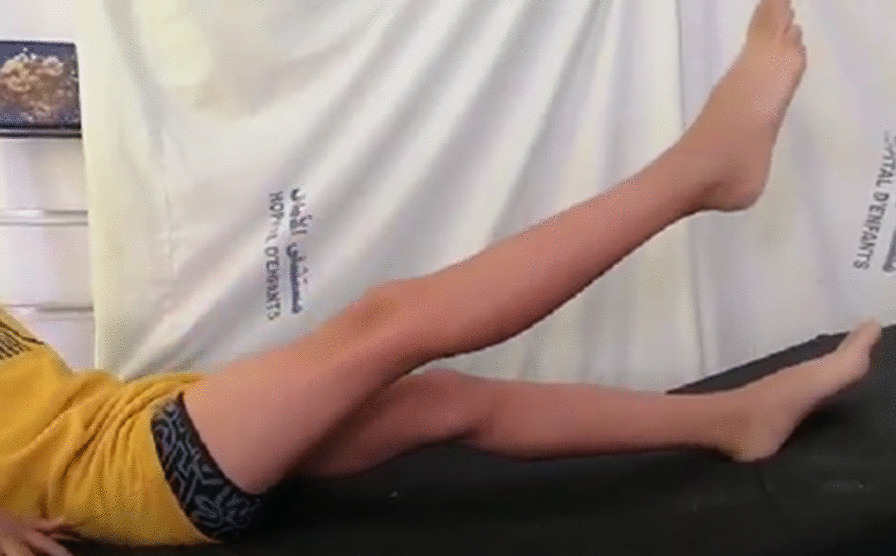
Active extension of the knee 6 years postoperatively

**Fig. 5:**
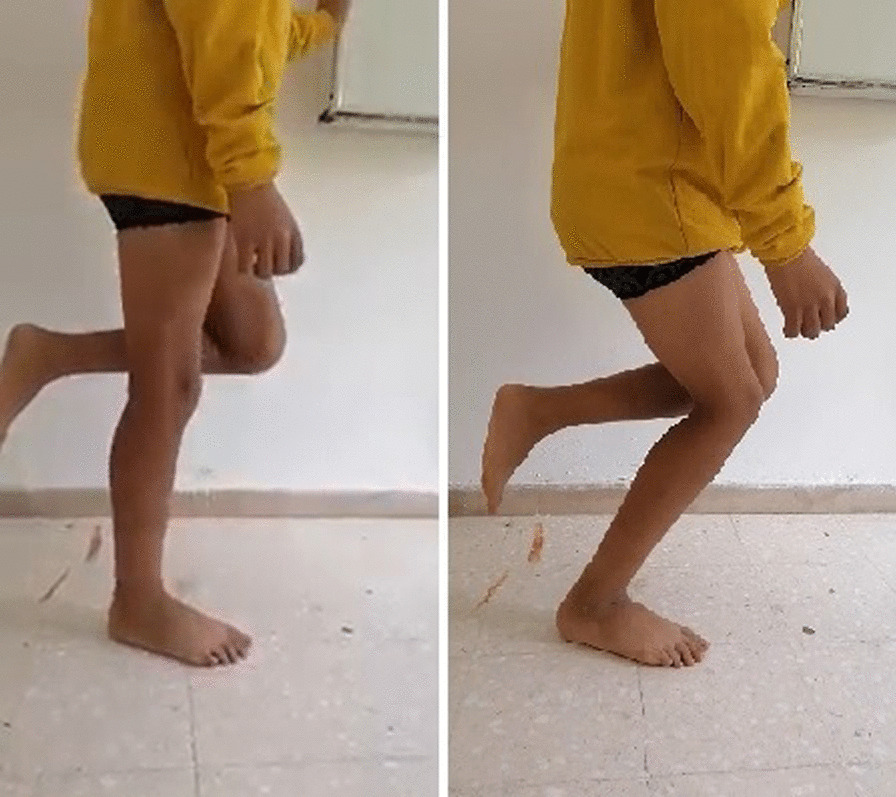
Postoperative single leg stance assessment

After closing the two wounds the knee was immobilized in extension with an above the knee cast for a 6 weeks period. After removal of the cast, the child was allowed to weight-bear and a passive and active physiotherapy performed with full flexion reached over a period of three months. At 6 years follow up, the child is reporting an improvement of the gait with a complete resolution of the right knee instability. On clinical examination, active range of motion was satisfying despite a persistent extension lag of 15° in the right knee (Fig. 4) and standing on one leg became possible (Fig. 5).

## Discussion

The clinical manifestations of the isolated PTLAH are very variable ranging from no complaint at all to flexion contractures of the knees in the neonatal period that impair the standing and walking ability of the patients [5]. In most cases, the patients complain from mild discomfort that minimally affects their physical activities [3].The chief complain in our patient was an instability of the knees more important in the right side with frequent falls and inability to practice sport. These manifestations were the same experienced by his father who has bilateral permanent laterally dislocated hypoplastic patella and was deemed by him sufficiently incapacitating to require some sort of intervention. The main physical finding was an extension lag of the knee that was much more important in the right side where the patella although hypoplastic and in a high position was present. In the left knee where the patella was totally absent the clinical manifestations were milder. It appears that the severity of the clinical manifestations does not depend on the presence or absence of the patella and that other mechanism may be involved.

The goal of the treatment in our patient was to obtain a strong active extension of the right knee by reconstructing the patellar tendon which was found to be replaced by a loose fibrous streak. Several different tendinous transfers have been described in the literature, the most common one being the semi-tendinous transfer [5–7]. We chose to use the semi-tendinous alongside the gracilis which have been harvested at the musculotendinous junction seen that both muscle have a medial distal insertion that can be kept intact providing a medial restraint to the extensor mechanism which has a natural tendency for lateral dislocation [7]. Furthermore, both muscles have a long tendinous portion and can easily be harvested using a tendon stripper. There’s no need to reroute the tendons to bring them in a more anterior position at the site of the natural distal insertion of the patellar tendon as it has been reported [5, 6]. The proximal insertion over the top of the patella with the folding back of the tendons provide a strong proximal anchor and a thick new patellar tendon. This technique is a modification of the semi-tendinous tenodesis described by Galeazzi for the treatment of the dislocation of the patella in skeletally immature patients [4]. We added here the transfer of the gracilis to obtain a thick new patellar tendon. After a follow up period of 6 years the right knee is symptoms free and the patient is asking to be operated on the left side which has milder symptoms.

## Conclusion

Reconstruction of isolated hypoplasia of the patella by a modified Galeazzi procedure is a safe and reliable technique for skeletally immature patients offering satisfying long-term outcomes.

## Data Availability

The data that support the findings of this article are available from the corresponding author, Mohamed Laroussi Toumia, upon reasonable request.

## Abbreviation

PTLAH: Patellar Aplasia Hypoplasia
